# Retinal Features and Preserved Vision in ENPP1 Deficiency: Long-Term Insights from Multimodal Imaging and Electrophysiology

**DOI:** 10.3390/ijms27188401

**Published:** 2026-09-21

**Authors:** Peter Heiduschka, Yvonne Nitschke, Christoph Clemens, Jens Storp, Nicole Eter, Frank Rutsch

**Affiliations:** 1 Department of Ophthalmology, Münster University Hospital, 48149 Münster, Germany; peter.heiduschka@ukmuenster.de (P.H.); dr.c.clemens@gmail.com (C.C.); jens.storp@gmail.com (J.S.); nicole.eter@ukmuenster.de (N.E.); 2 Department of General Pediatrics, Münster University Children’s Hospital, 48149 Münster, Germany; yvonne.nitschke@ukmuenster.de; 3 INTEC Network of Ectopic Calcification, Center for Medical Genetics Ghent, Corneel Heymanslaan 10, 9000 Ghent, Belgium

**Keywords:** ENPP1 deficiency, ectopic calcification, optical coherence tomography, electroretinography

## Abstract

Loss-of-function variants in ectonucleotide pyrophosphatase/phosphodiesterase 1 (*ENPP1*) impair pyrophosphate homeostasis, leading to pathological ectopic calcification. We present the first long-time longitudinal single-case ophthalmic assessment of a male patient with ENPP1 deficiency. Multimodal evaluations included spectral-domain optical coherence tomography (OCT), OCT angiography (OCT–A), infrared scanning laser ophthalmoscopy (IR-SLO), fundus autofluorescence, fluorescein angiography, dark adaptometry, full-field and multifocal electroretinography (ERG), and the measurement of visual evoked potentials (VEP). All data were compared to normative data obtained from age-matched controls in our clinic. Visual acuity declined slightly from 1.0 to 0.7 (OD) and 0.63 (OS) within ten years. IR-SLO revealed numerous retinal speckles without structural OCT correlates or progression. OCT showed subtle changes in layer-specific retinal thickness, without reaching significance compared to healthy controls. OCT–A revealed reduced flow densities in superficial and deep capillary plexuses and preserved foveal avascular zone metrics. Scotopic ERG and multifocal ERG amplitudes were reduced and some ERG implicit times increased. Adaptometry showed a reduced rod sensitivity. Despite systemic ectopic calcification, retinal structure and function remained largely preserved. The findings suggest mild subclinical retinal involvement and support the use of electrophysiology as candidate noninvasive biomarkers for monitoring. Distinct from ABCC6-related PXE, *ENPP1* deficiency may exhibit a slower, more stable retinal course.

## 1. Introduction

Ectonucleotide pyrophosphatase/phosphodiesterase 1 (*ENPP1*) is a key regulator of extra-cellular nucleotide metabolism and tissue mineralization. Loss-of-function variants in *ENPP1* lead to a spectrum of autosomal recessive disorders characterized by ectopic calcification and disturbed phosphate homeostasis [1,2]. The most severe phenotype is generalized arterial calcification of infancy (GACI), while milder forms include autosomal recessive hypophosphatemic rickets type 2 (ARHR2), which typically presents later with bone pain, rickets, and short stature [3,4]. In both conditions, pathogenic *ENPP1* variants cause a reduction of extracellular pyrophosphate (PPi), a potent inhibitor of hydroxyapatite deposition, thereby facilitating aberrant calcification of soft tissues, including vasculature.

While systemic features of *ENPP1* deficiency are well documented, ocular manifestations remain poorly characterized. Intriguingly, recent studies have highlighted phenotypic overlaps with pseudoxanthoma elasticum (PXE), a related calcification disorder caused by mutations in *ABCC6*. PXE typically affects Bruch’s membrane, leading to angioid streaks, choroidal neovascularization, and visual impairment [5]. Previously, we demonstrated that GACI individuals with *ENPP1* deficiency who survive infancy may develop late-onset PXE-like skin and ocular findings. Additionally, we identified *ABCC6* variants in patients diagnosed with GACI who did not carry variants in *ENPP1*, suggesting a shared pathomechanism of ABCC6 and *ENPP1* deficiency, involving impaired extracellular PPi homeostasis [6]. This mechanism was later proven for ABCC6 deficiency, where it was shown that failure of nucleotide release in the liver resulted in low extracellular PPi levels in ABCC6 deficiency [7,8].

The clinical and mechanistic parallels of ABCC6 and *ENPP1* deficiency are underscored by murine models. ENPP1-deficient mice (strain asj) exhibit ectopic mineralization of multiple tissues, including the dermal sheath of vibrissae and the eye, mimicking both GACI and PXE phenotypes [9], like Abcc6-deficient mice [10]. In this respect, Bruch’s membrane calcification associated with functional impairment of the retina has been recently described in an additional ENPP1-deficient animal model (ARVO abstracts [11,12]).

Furthermore, in a prospective cohort study, Ferreira et al. identified ocular changes consistent with PXE—including peau d’orange and angioid streaks—in individuals with *ENPP1* variants, underscoring the need for dedicated ophthalmic surveillance in these patients [13]. In parallel, on the other side, detailed phenotyping studies in ABCC6-deficient PXE patients provided valuable insight into comparable risk patterns, which may be applied in *ENPP1* deficiency [5,14].

Despite these emerging insights, data on the longitudinal ophthalmic course in *ENPP1* deficiency are scarce, and, to our knowledge, no studies have employed comprehensive multimodal retinal imaging or functional testing. Given the role of Bruch’s membrane calcification in visual decline in PXE, analogous processes may also threaten vision in ENPP1-deficient individuals.

In this study, we report a 10-year longitudinal follow-up of a patient with molecularly confirmed *ENPP1* deficiency. Using a combination of spectral-domain OCT, OCT angiography, IR-SLO, and full-field and multifocal ERG, we detail subtle yet stable structural and functional changes and differences compared to an age-matched healthy control group. Our findings provide a deeper understanding of the ocular phenotype in *ENPP1* deficiency and support the use of retinal imaging and electrophysiology in order to define noninvasive biomarkers for long-term disease monitoring, such as the integrity and thickness of retinal layers, flow density, and/or amplitudes obtained in electrophysiological recordings.

## 2. Results

### 2.1. Case Presentation

The male proband was born via cesarean section at 36 weeks of gestation due to fetal distress (birth weight 3960 g; APGAR 9/10/10) to consanguineous Turkish parents (second-degree relatives). The pregnancy was complicated by gestational diabetes controlled with insulin. Family history included multiple pregnancy losses, but three siblings were healthy.

On day 5 of life, the neonate developed tachypnea, cyanosis, and severe lactic acidosis. A chest X-ray revealed cardiomegaly; echocardiography showed patent ductus arteriosus, foramen ovale, and severely reduced ventricular contractility, leading to a diagnosis of congestive heart failure due to dilated cardiomyopathy. After 19 days of mechanical ventilation, he stabilized. Initial metabolic and mitochondrial workup was unremarkable.

Subsequent management included digoxin, diuretics, and spironolactone. By six months of age, the proband developed severe arterial hypertension (up to 120/80 mm Hg), and cardiac catheterization showed a hypertrophied left ventricle, mitral regurgitation, patent ductus arteriosus, hypoperfusion of the upper left lung lobe, and bilateral renal artery stenoses. The ductus arteriosus was surgically ligated, and antihypertensive treatment was intensified with captopril, prazosin, and nifedipine.

At 8 months, the patient presented with painful swelling of both wrists. An X-ray revealed peri-articular stippled calcifications in the carpal and ankle joints. A biopsy of the temporal artery confirmed GACI with calcification of the lamina elastica interna [15]. Imaging showed widespread vascular calcifications of the cardiac septum, aorta, carotid, and celiac arteries, though brain MR angiography was normal.

Low urinary inorganic PPi levels [15], low *ENPP1* plasma enzymatic activity, and plasma concentration suggested a loss of function of *ENPP1* [16], which was confirmed by molecular genetic testing. Mutation analysis of the *ENPP1* gene revealed the truncating variant c.2677G>T; p.E893* on both alleles (family 1 [17]).

Treatment with oral etidronate (13–20 mg/kg/day) was initiated and maintained for 8 years. During this time, the patient showed mild psychomotor delay and recurrent respiratory infections. Left ventricular hypertrophy persisted with an ejection fraction of 21–50%, and arterial hypertension required continued multi-drug therapy. Etidronate treatment was associated with a modest regression in periarticular calcifications, but renal artery stenoses remained unchanged. At the age of 8 years, angioplasty of both renal arteries resulted in a reduction of renal arterial stenosis. Furthermore, angiography revealed a complete occlusion of the internal carotid arteries bilaterally with extensive collateralization via the external carotid arteries, while it was not clear whether this occlusion was associated with calcification. Because of genu varum, the patient received eight plates for guided growth surgery of the right leg at the age of 14 years.

At the age of 15 years, the patient presented with recurrent dizziness and syncope. An electrocardiogram revealed a second-degree atrioventricular (AV) block, and Holter monitoring demonstrated episodes of complete (third-degree) AV block. A CT scan showed the calcification of the interventricular septum involving the region of the cardiac conduction system, sparing the coronary arteries. A transvenous pacemaker was inserted, resulting in the resolution of syncopal symptoms. However, at the age of 19 years, he experienced acute cardiac decompensation, renal failure and pulmonary edema in the course of gastroenteritis, necessitating intensive care treatment leading to complete recompensation.

The cardiac status of the proband is regularly monitored, and he is currently treated with colchicine, eplerenone, bisoprolol, ramipril, torasemide, edoxaban, simvastatin, allopurinol and vitamin D3 (cholecaliferol). Because of sensorineuronal hearing impairment diagnosed at the age of 18 years, he is wearing hearing aids.

Plasma PPi levels remained continuously low in the proband, ranging from 0.04 to 0.2 µM (normal range for adults (99% confidence limits): 0.16–3.40 µM/L)) [18].

At the age of 19 years, he first presented to the ophthalmology department for routine screening by multimodal imaging. Since then, he has been followed regularly by multimodal imaging and functional testing. The proband is currently 29 years old; he is ambulatory, and his heart failure is in NYHA stage II, characterized by mild symptoms and a mild limitation in his ability to engage in physical activity. He is employed by a sheltered workshop.

### 2.2. Ophthalmological Status

The best corrected visual acuity was at 1.0 on both eyes until 2016. Afterwards, it showed a decline to 0.8 (2018) and reached 0.7 (OD) and 0.63 (OS) in 2024. Intraocular pressure was in the normal range, between 15 mm Hg and 19 mm Hg.

**Figure 1:**
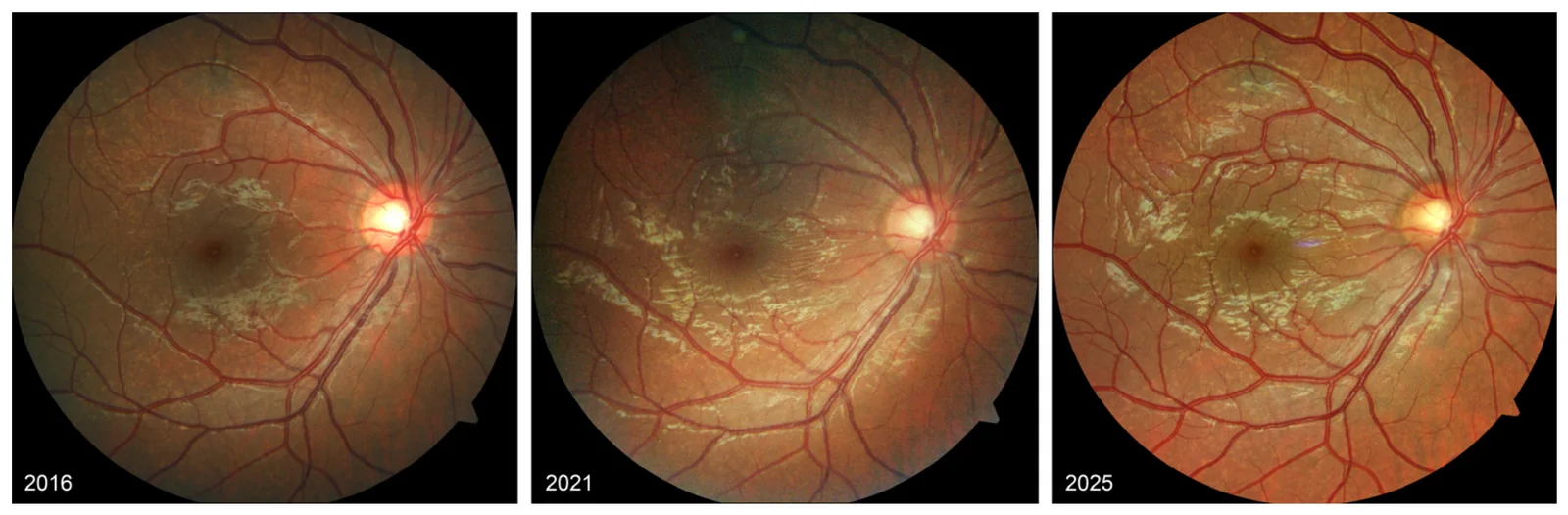
Color fundus images of the right eye at time points indicated. No changes of the normal appearance can be seen within nine years.

In the anterior part of the eye, normal status of the cornea and anterior chamber could be stated. There were some vacuoles in the lenses, which, apart from that, were clear. The pupil showed normal behavior. A normal optic nerve head and macula were seen in fundus color images (Figure 1). In the retina, a normal vasculature was present, without bleeding or vascular streaks. Some diffuse hypopigmented areas were seen in the regions of the big retinal vessels.

**Figure 2:**
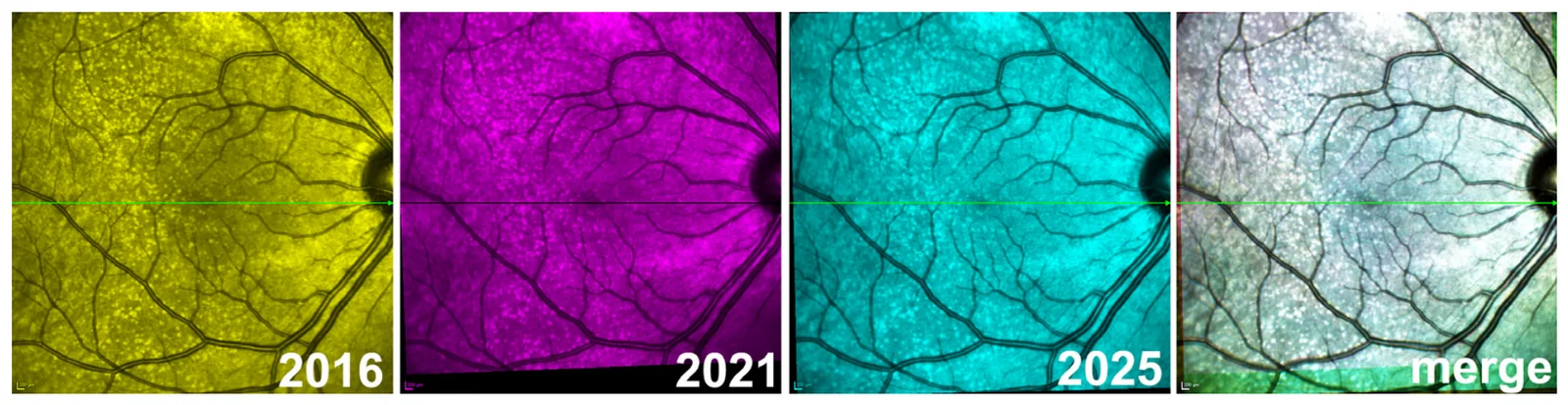
Positions and number of speckles visible in the IR-SLO images did not change over nine years, as demonstrated by the merge of the colorized images.

Numerous bright speckles were seen in the IR-SLO images. We did not see a change in their size, number or distribution over the time span of our examinations (Figure 2). No corresponding structural abnormalities were detected in OCT scans. Moreover, the speckles were not apparent on fundus autofluorescence imaging.

Fluorescein angiography did not show any abnormal findings compared to the healthy controls. One example of a completely normal image is shown in Supplemental Figure S1.

### 2.3. Retinal Structure

**Figure 3:**
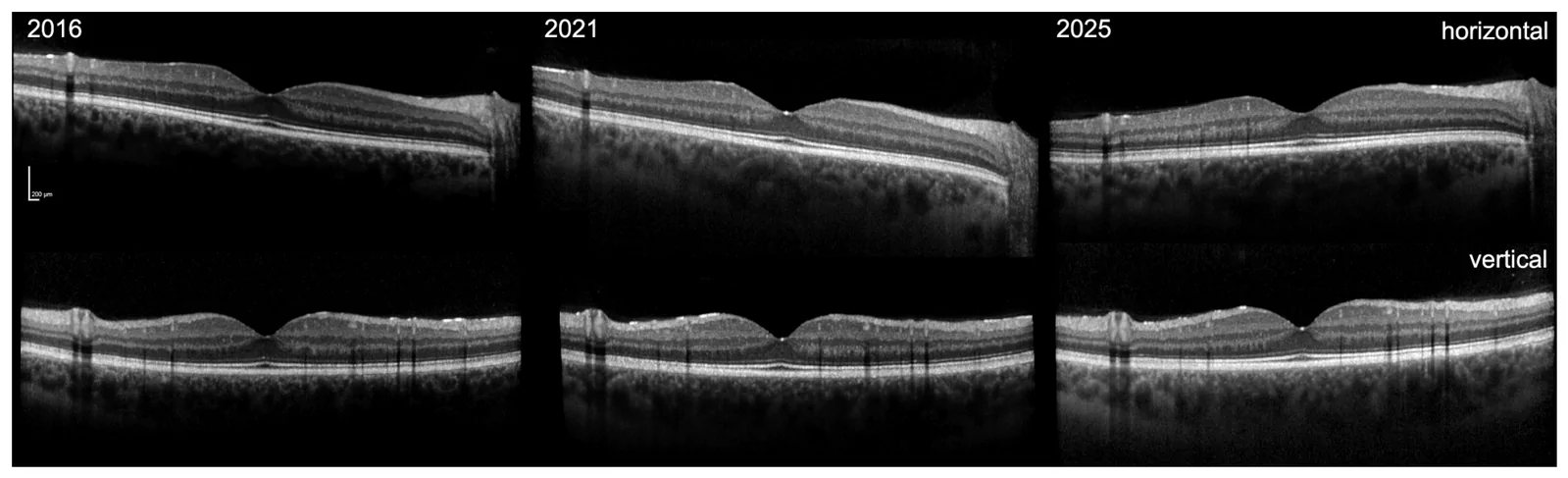
Macular OCT scans obtained within nine years, as indicated.

Macular OCT images did not change noticeably during the observation time (Figure 3). They did not show any obvious irregularities except for the band of the interdigitation zone that appeared to be “melted” with the RPE band in the images.

**Figure 4:**
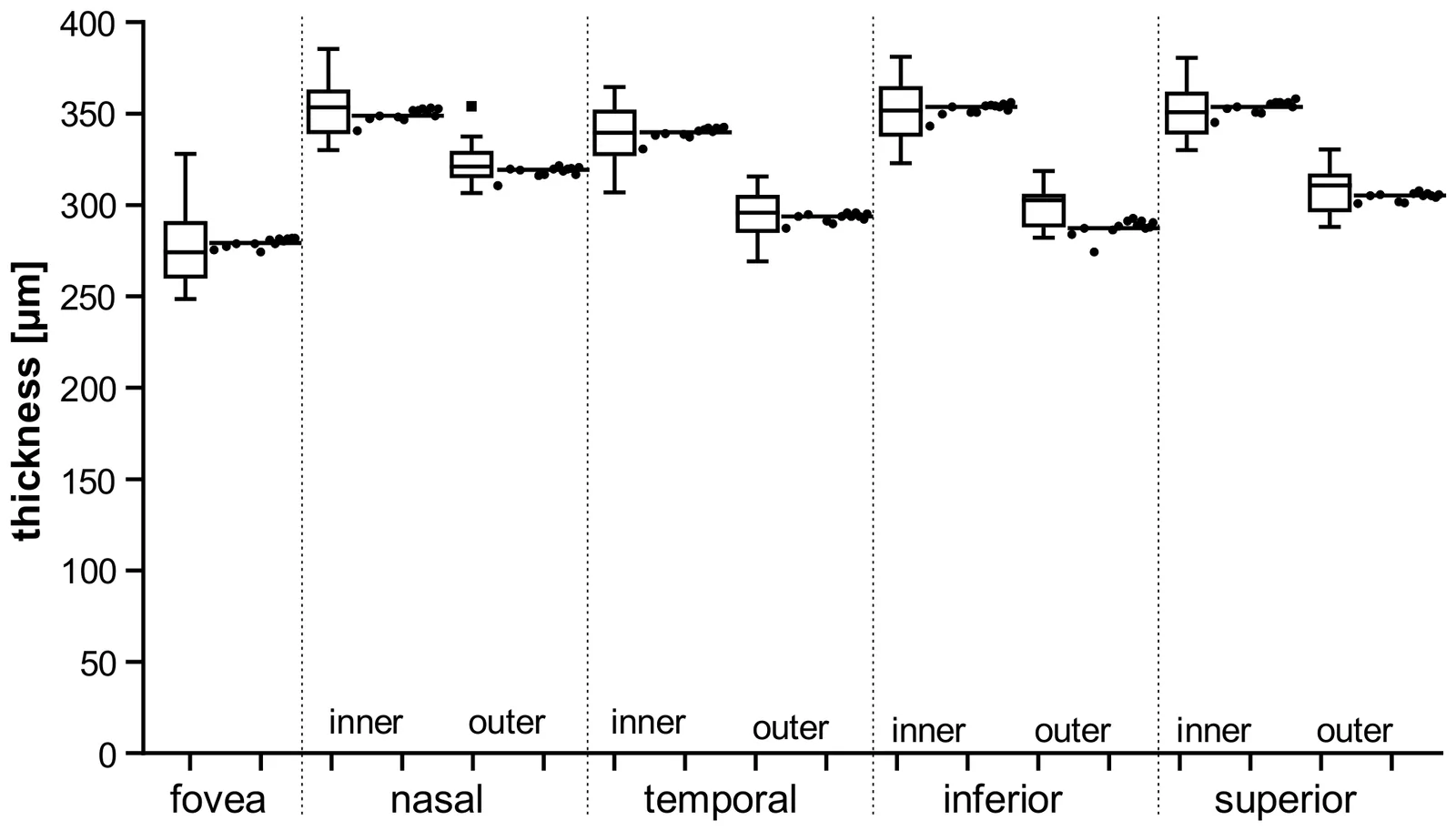
Comparison of retinal thickness between age-matched controls (*n* = 14) and the patient in the single sectors of the ETDRS grid, as indicated. Range of controls used in this study is indicated by Tukey box plots, and patient data are shown by dots, aligned by time points of measurement between 2016 and 2026. The horizontal lines between the dots indicate medians of the data.

We measured the thicknesses of the retina and retinal layers in the patient’s eyes in the OCT images at different time points from 2016 to 2026. There was a subtle increase of retinal thickness during the observation time, depending on the sector of the Early Treatment Diabetic Retinopathy Study (ETDRS) grid checked. Total retinal thickness did not differ significantly from the age-matched controls (Figure 4).

**Figure 5:**
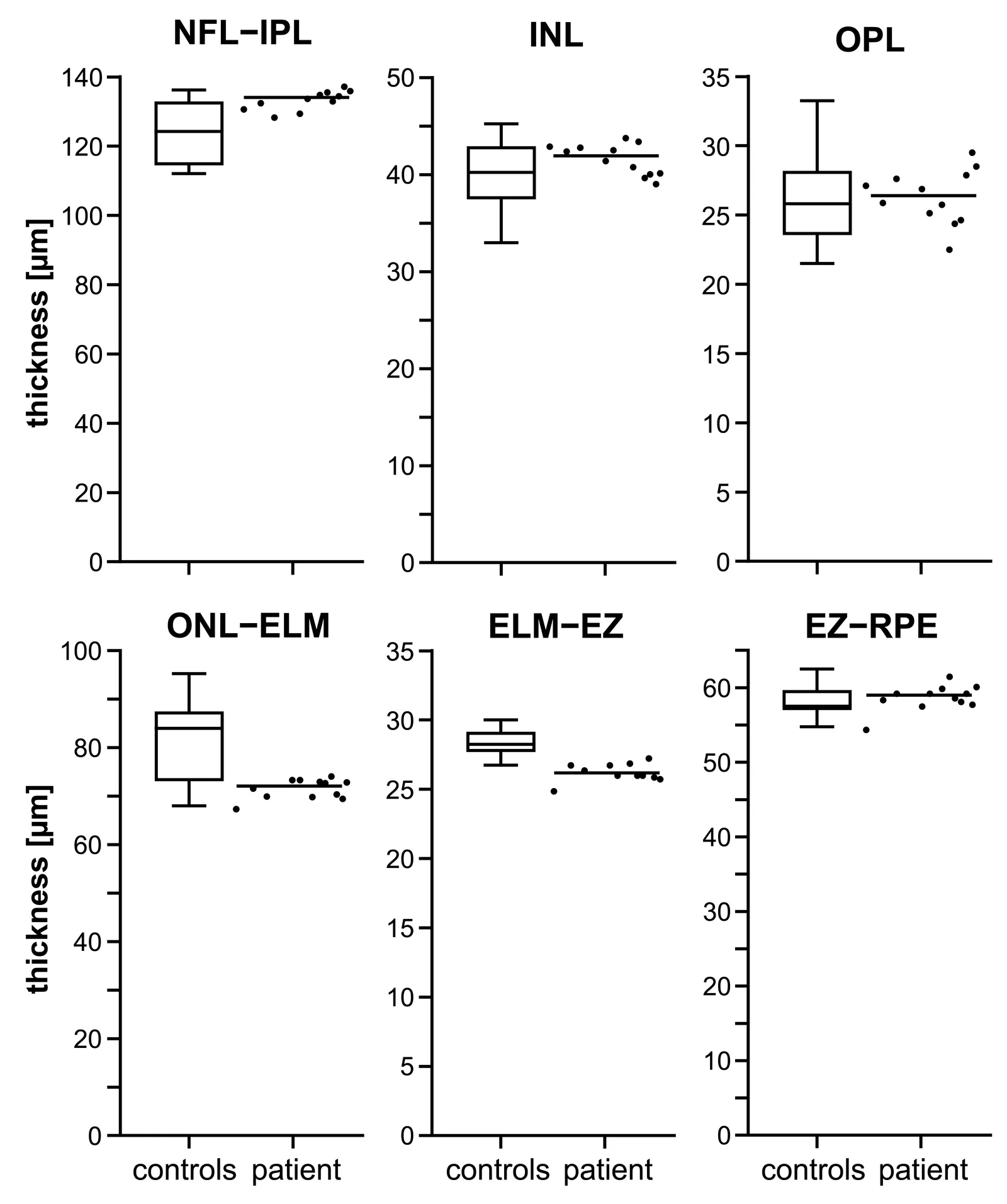
Comparison of thickness of retinal layers between age-matched controls (*n* = 14) and the patient as indicated. Range of controls used in this study is indicated by Tukey box plots, and patient data are shown by dots, aligned by time points of measurement from 2016 to 2026. The short horizontal lines between the dots indicate medians of the data. Please note the different scales.

We then manually checked the thicknesses of different retinal layers in the OCT images, as described in Section 4. No significant differences between the patient and the controls were found in the thicknesses of the retinal layers, as depicted in Figure 5, i.e., nerve fiber layer, ganglion cell layer and inner plexiform layer (NFL–IPL), inner nuclear layer (INL), outer plexiform layer (OPL), thickness of the outer nuclear layer to the external limiting membrane (ONL-ELM), distance between the ELM and the ellipsoid zone (ELM–EZ) as well as distance between the ellipsoid zone (EZ), and the retinal pigment epithelium (RPE). Similar to the data shown in Figure 4, we also found a certain change in the thickness of the retinal layers (Figure 5). While thickness decreased slightly in the INL, it showed a slight increase in all other layers.

**Figure 6:**
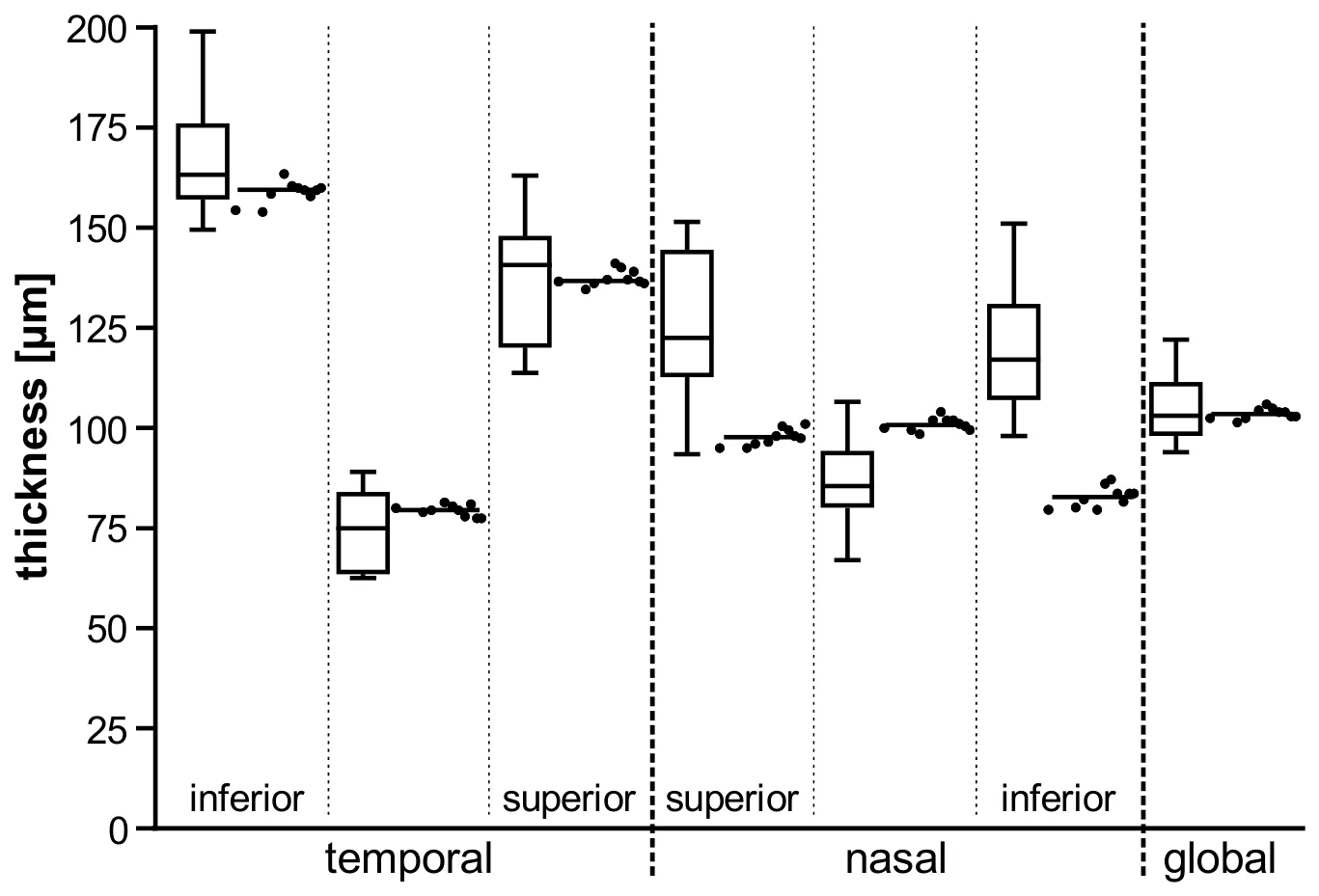
Comparison of thicknesses in age-matched controls (*n* = 11) and the patient of retinal NFL, displayed by the sectors of the measurement grid as indicated. Range of controls used in this study is indicated by Tukey box plots, and patient data are shown by dots and aligned by time points of measurement between 2018 and 2026. The horizontal lines between the dots indicate medians of the data.

The retinal nerve fiber layer (NFL) thickness was measured at the Bruch’s membrane opening (BMO) between 2018 and 2026. For an example, see Supplemental Figure S2. The global thickness was within the normal range of age-matched controls and as provided by the internal database provided by the Heidelberg Engineering software HEYEX 2.0. Nevertheless, we saw deviations of retinal NFL thickness in the nasal sectors, where the retinal NFL thickness was smaller in the inferior and superior sectors and bigger in the nasal sector compared to the controls (Figure 6).

**Figure 7:**
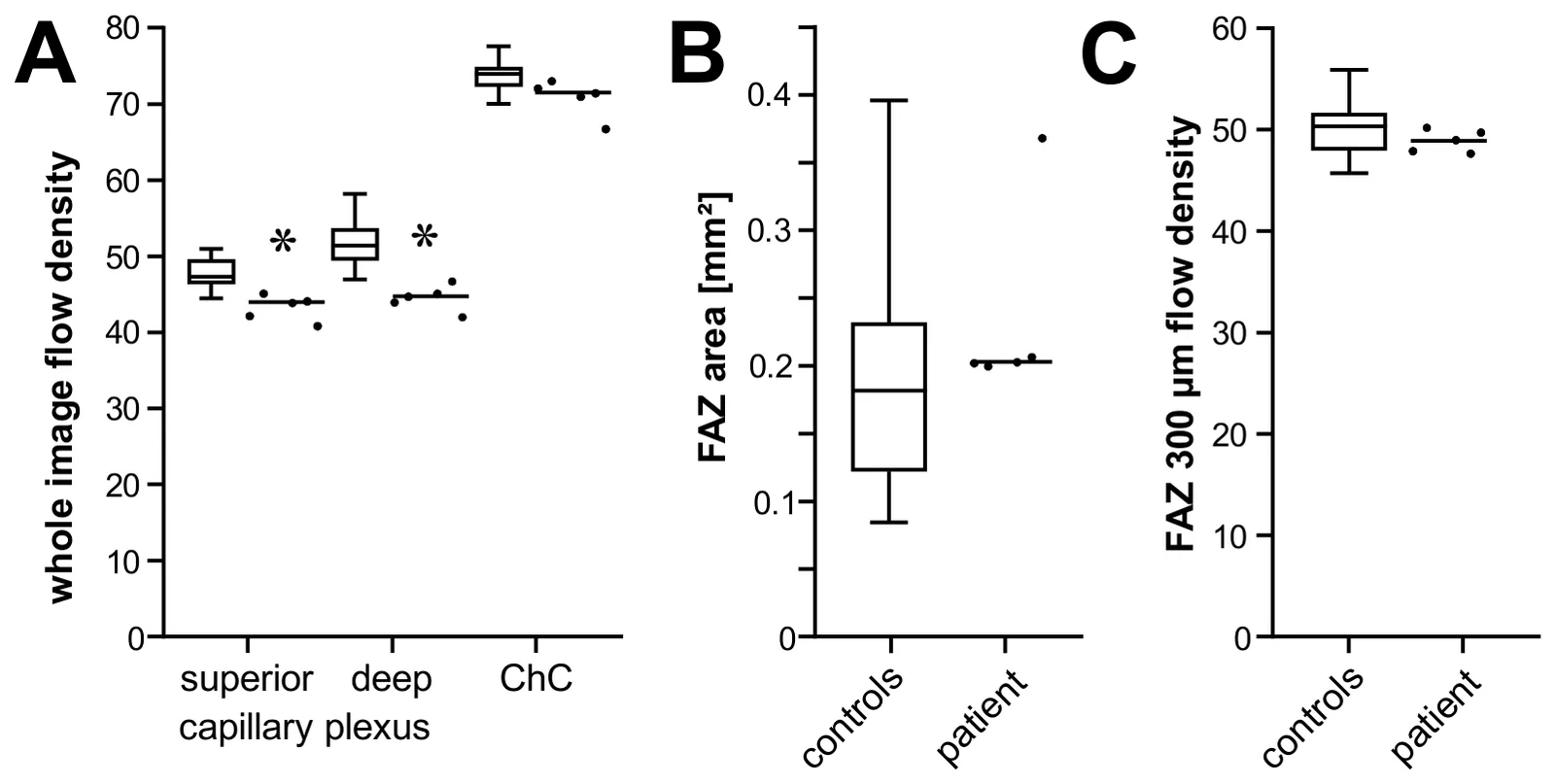
Comparison of OCT–A parameters between age-matched controls (*n* = 29) and the patient. (**A**) Vessel densities as indicated. (**B**) Area of the foveal avascular zone (FAZ). (**C**) FAZ 300 µm flow density. Range of controls used in this study is indicated by Tukey box plots, and patient data are shown by dots, aligned by time points of measurement from 2023 to 2026. The horizontal lines between the dots indicate medians of the data. Significance of differences between controls and the patient are shown by an asterisk: * *p* < 0.05.

We also performed imaging with optical coherence tomography angiography (OCT–A). No obvious abnormal findings were seen in the OCT–A images (Supplemental Figure S3). Results of evaluation of the OCT–A images are shown in Figure 7. Retinal flow densities in the superficial capillary plexus and deep capillary plexus showed lower values than in age-matched controls, and the difference was significant (Figure 7A). Flow density in the choriocapillaris (ChC) showed a smaller difference to the controls. Vessel densities in the three layers do not change very much, although the last measurement seemed to show a slight decrease (Figure 7A). We found an increased area of the foveal avascular zone (FAZ) in the last measurement (Figure 7B), no changes in the 300 µm flow density of the FAZ (Figure 7C), and no significant difference to control values.

### 2.4. Visual Function

**Figure 8:**
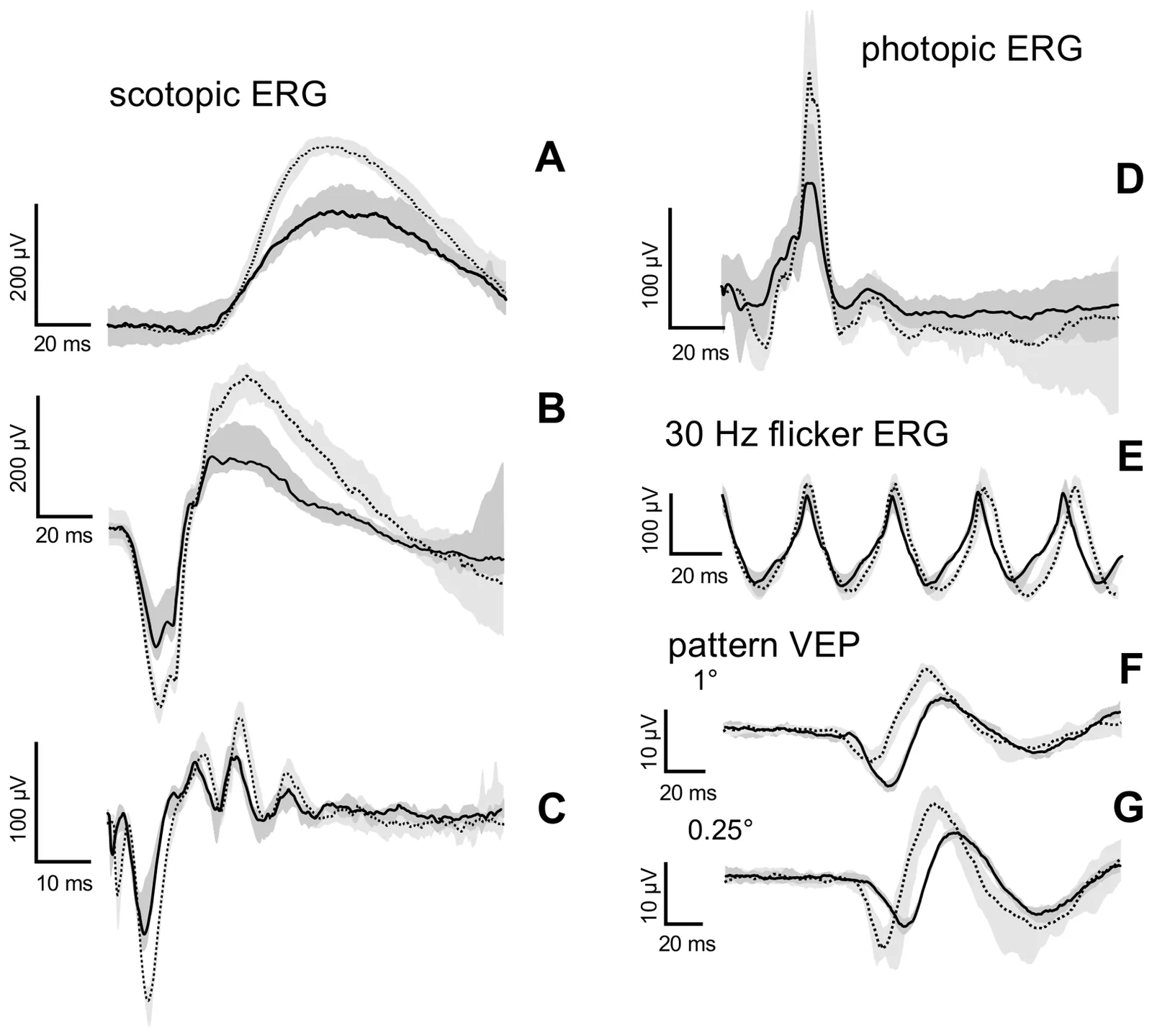
Comparison of waveforms obtained by electrophysiological measurements in the patient and the controls. Shown are the medians of all data obtained in the patient (solid lines) and in 11 age-matched controls (dotted lines). The shaded areas represent the range of 25th percentile to 75th percentile of the data of both the patient (dark gray) and the controls (light gray). (**A**) Scotopic flash ERG at 0.01 cd·s/m^2^, (**B**) scotopic flash ERG at 3.0 cd·s/m^2^, (**C**) scotopic oscillatory potentials at 100 cd·s/m^2^, (**D**) photopic flash ERG at 3.0 cd·s/m^2^, (**E**) photopic 30 Hertz flicker ERG at 3.0 cd·s/m^2^, (**F**) pattern VEP recording with 1° check size, (**G**) pattern VEP recording with 0.25° check size.

Visual function was evaluated by electroretinography (ERG), measurement of visual evoked potentials (VEP), and dark adaptometry. We performed ERG and VEP measurements eight times between 2021 and 2026, and some ERG measurements already in 2013. In Figure 8, we show the median waveforms of these examinations compared with the median waveforms of healthy controls.

**Figure 9:**
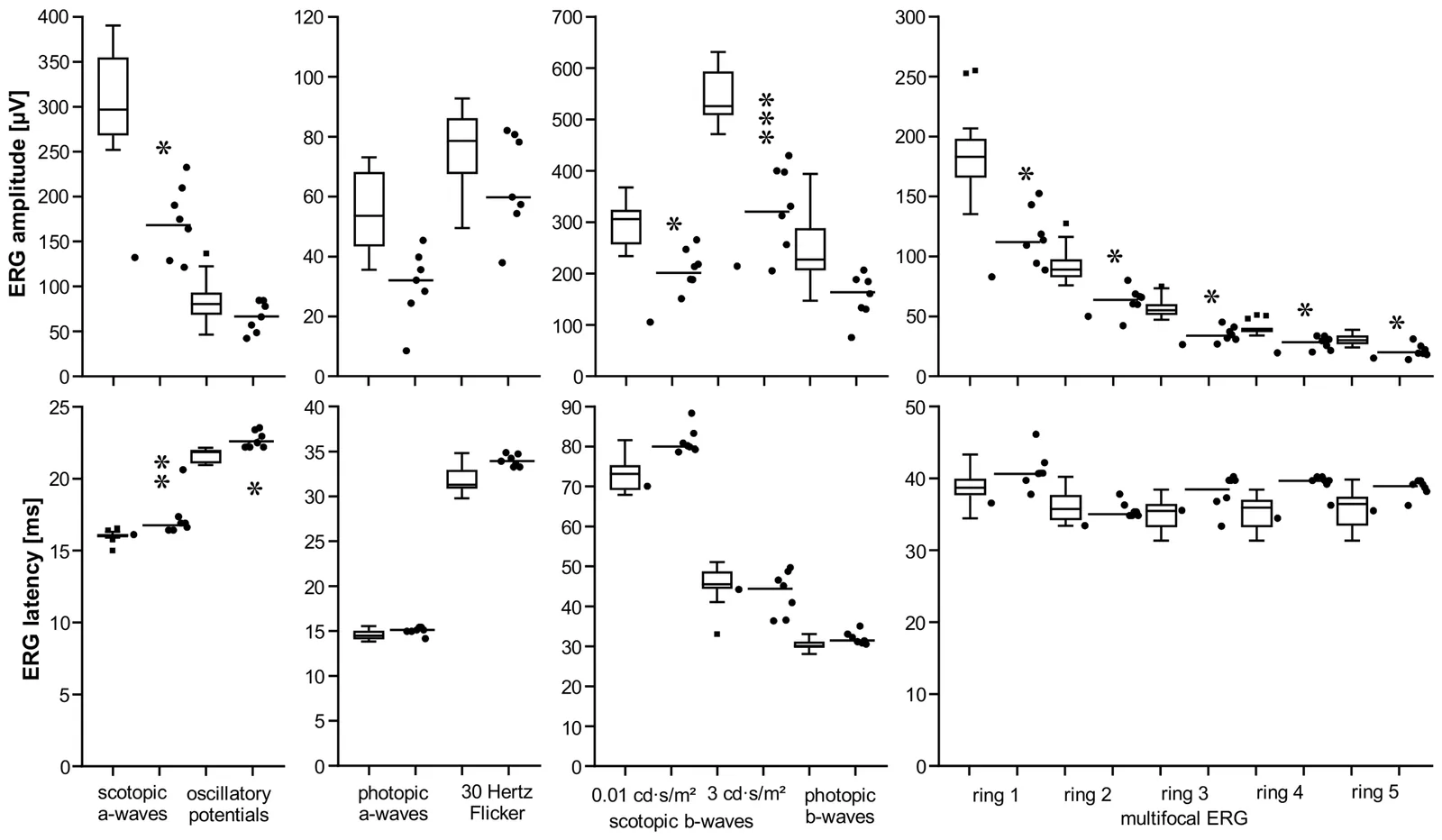
Comparison of ERG amplitudes and latencies between the patient and age-matched controls (*n* = 17 for flash ERG and *n* = 18 for multifocal ERG), as indicated. Range of controls used in this study is indicated by Tukey box plots, and patient data are shown by dots, aligned by time points of measurement between 2013 and 2026. The horizontal lines between the dots indicate medians of the data. Significance of differences between controls and the patient is shown by asterisks: * *p* < 0.05, ** *p* < 0.01, *** *p* < 0.001.

**Figure 10:**
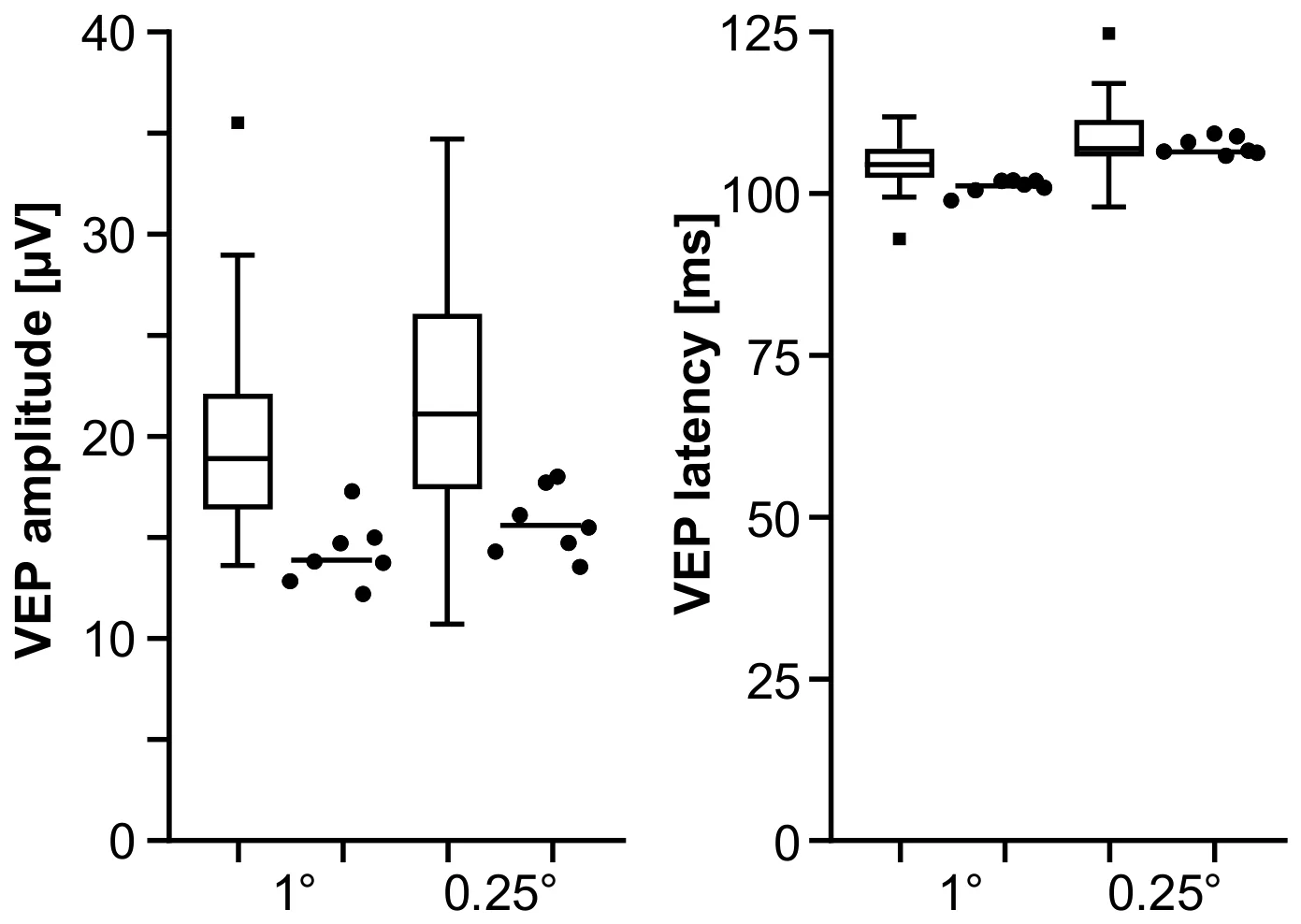
Comparison of VEP amplitudes and latencies between the patient and age-matched controls (*n* = 41), as indicated. Range of controls used in this study is indicated by Tukey box plots, and patient data are shown by dots, aligned by time points of measurement between 2021 and 2026. The horizontal lines between the dots indicate medians of the data.

The exact results of data evaluation are shown in Figure 9 and Figure 10. When compared to age-matched normative data obtained in our clinic, we found significantly reduced amplitudes of electroretinographic waves in both scotopic and photopic multifocal modes. The latencies were slightly prolonged. The amplitudes and latencies of VEP were in the lower and normal range of normative values, respectively. The averaged values of amplitudes and latencies are shown in Supplemental Tables S1–S3, and the values of age-matched normative data are given for comparison.

**Figure 11:**
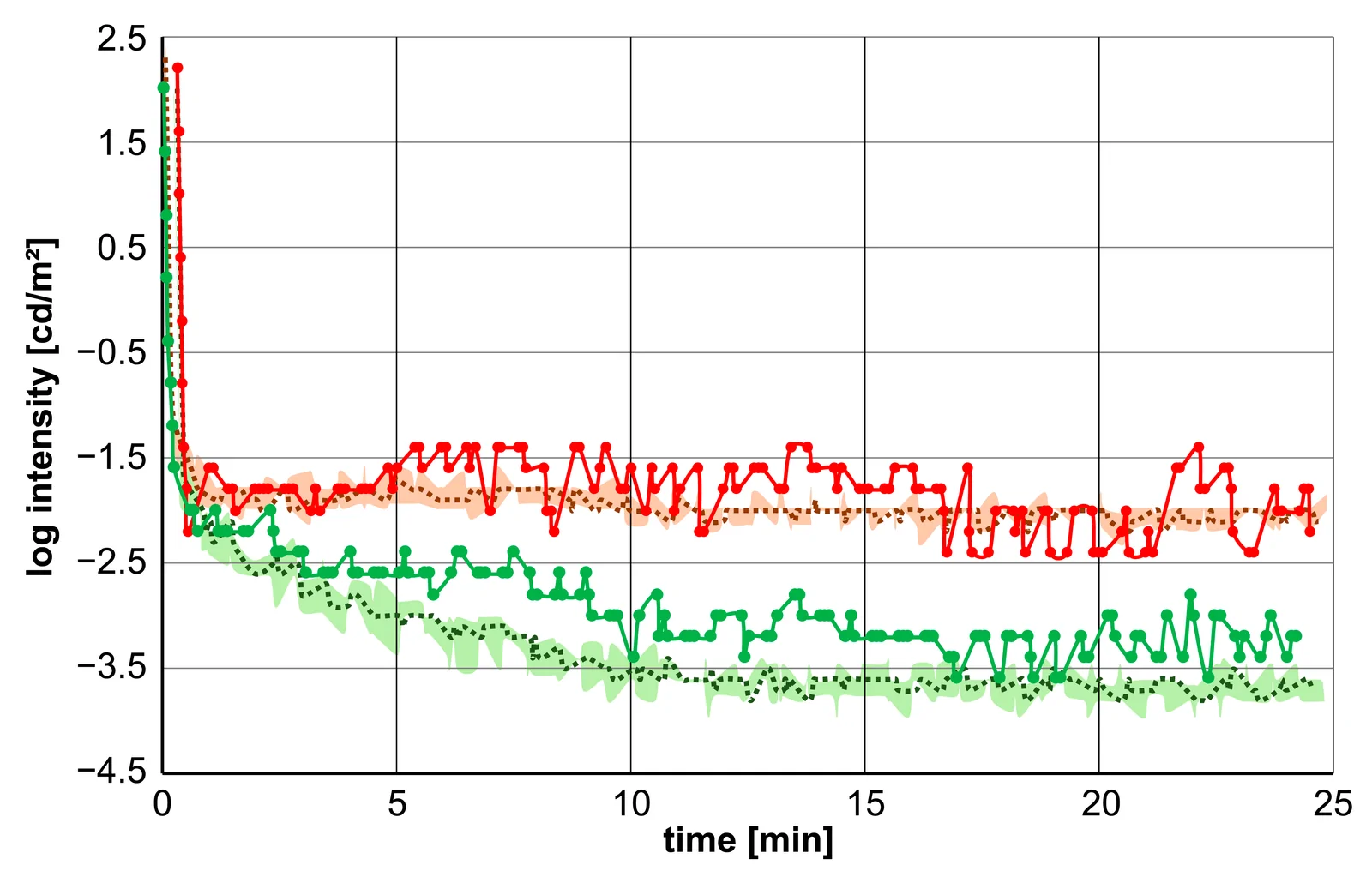
Comparison of dark adaptometry examination results in the patient and the controls, separated by responses of cones (red) and rods (green). Shown are the medians of the data obtained in the patient (bright red or green lines with dots) and in the age-matched controls (*n* = 6) (dotted dark red or green lines). The light red and light green areas represent the range of 25th percentile to 75th percentile of the data obtained in the controls.

So far, we were able to perform dark adaptometry only once on the patient (year 2026). The result is shown in Figure 11. The patient reached approximately the same cone sensitivity as the controls, although there were clear fluctuations in the recorded sensitivity during the measurement. Rod sensitivity was clearly smaller than in the controls after more than 15 min of dark adaptation and came closer to the control values only after 17 min, without reaching them completely. It should be emphasized that the results shown in Figure 11 apply specifically to the modified protocol in this study.

## 3. Discussion

This study provides the first longitudinal, multimodal retinal imaging and functional assessment of a patient with *ENPP1* deficiency, offering unprecedented insight into the natural history and subclinical ocular manifestations of this rare systemic calcification disorder. While it is not yet clear how calcification may contribute to them, we observed subtle but consistent structural and functional retinal changes in the absence of overt visual decline over a follow-up period of ten years. These findings suggest that retinal changes may provide potential early biomarkers and could contribute to monitoring disease progression in *ENPP1* deficiency.

Mechanistically, our results align with prior work, demonstrating that *ENPP1*, like ABCC6, plays a critical role in maintaining extracellular PPi homeostasis. PPi is a key inhibitor of pathological mineralization, and its deficiency leads to ectopic calcification, as seen in both generalized arterial calcification of infancy (GACI) and pseudoxanthoma elasticum (PXE) [6,19]. Biallelic mutations in *ENPP1* not only result in early-life vascular calcification but may also produce late-onset PXE-like ocular and cutaneous features [13]. These parallels are supported by shared histopathological hallmarks—possibly also calcification of Bruch’s membrane—underscoring the concept of a common molecular pathomechanism involving PPi deficiency as an important inhibitor of calcification in elastic tissues.

Despite these mechanistic similarities, our patient displayed remarkably stable retinal morphology on spectral-domain OCT over nearly a decade and only a slight loss of visual acuity. The IR-SLO speckles, though consistently observed (Figure 2), lacked a defined structural correlate and remained unchanged over time, suggesting a subclinical retinal phenotype. Retinal thickness was in the control range in most cases and showed just a slight increase over the years (Figure 5 and Figure 6). The mechanism underlying these thickness changes remains uncertain.

Flow densities in the superficial and deep capillary plexuses were significantly smaller than in the control group in our patient. Functional testing by scotopic full-field and multifocal ERG revealed reduced amplitudes with a slight delay in implicit times in most cases, suggesting subthreshold electrophysiological deviations from age-matched normative ranges without frank degeneration. However, ERG amplitudes showed quite unstable behavior and a trend to increase over the course of some years, while being smaller again during the last session, which we cannot explain at the moment. In summary, ERG measurements reveal an abnormally variable retinal function in our patient, with scotopic amplitudes significantly smaller than control values at many visits. We also found a reduced rod sensitivity in the dark adaptometry examination, which goes in line with the smaller scotopic ERG amplitudes. However, additional dark adaptometry examinations will be needed on longitudinal follow-up in order to substantiate the finding of impaired rod sensitivity. Such a reduction of adaptometric rod sensitivity was also reported in PXE patients [5,20].

These findings collectively indicate a mild and slowly progressive retinal involvement in *ENPP1* deficiency that differs in severity and pace from the retinal pathology typically observed in ABCC6-related PXE. PXE is characterized by a well-defined sequence of ocular changes that begin with subretinal punctate accumulations known as “peau d’orange”, followed by the development of angioid streaks—cracks in Bruch’s membrane radiating from the optic disk. Over time, patients may develop extensive calcification of Bruch’s membrane, which can lead to progressive chorioretinal atrophy, reduced retinal sensitivity, and choroidal neovascularization. These complications are responsible for a significant decline in visual acuity, particularly from the second to fourth decades of life [14,21]. Fundus autofluorescence often reveals widespread signal alterations even before clinical symptoms emerge, and dark adaptation is frequently impaired. In advanced stages, OCT imaging shows the collapse and thinning of outer retinal layers, often with disruption of the RPE and ellipsoid zone, leading to irreversible central vision loss (for a review, see Ref. [5]).

In contrast, the ENPP1-deficient retina in our case showed none of these clear hallmark signs of degeneration, indicating a delayed progression and/or a fundamentally less aggressive retinal phenotype in this patient. This contrast may reflect differences in local calcification dynamics, tissue-specific enzyme activity, or other modifying factors. The causes of reduced ERG amplitudes and reduced rod sensitivity remain unclear. In the patient, the outer nuclear layer was thinner than in the controls, and flow densities of both superficial capillary plexus and deep capillary plexus were significantly smaller. Whether these structural and microvascular deviations are sufficiently pronounced to contribute to the reduced ERG amplitudes and their intervisit variability remains uncertain. Another potential contributing factor could be the calcification of the retinal arteries. However, such calcification usually does not occur in elastic arteries that are present in the retina, whereas it could occur in choroidal arteries or the choriocapillaris. Unfortunately, imaging techniques available in our clinic so far do not allow for direct visualization of potential calcifications in the retina or in the choroid. The same is true for Bruch’s membrane, which could not be investigated in the patient. In summary, in this patient, the retinal findings remained relatively mild and largely stable during the observation period; however, the exploratory single-case design precludes conclusions regarding differences in retinal disease course between *ENPP1* deficiency and ABCC6-related PXE.

The novelty of this study lies in the integration of long-term structural and functional retinal data from a genetically confirmed ENPP1-deficient patient, highlighting candidate retinal measures that warrant further evaluation as potential noninvasive biomarkers in larger cohorts of patients with *ENPP1* deficiency. To our knowledge, this is the first report to integrate quantitative OCT layer analysis, OCT angiography metrics, and serial ERG measurements in this patient population. Further studies with more ENPP1-deficient patients must show to what extent our findings may be generalized.

From a translational perspective, these results highlight the potential of multimodal retinal assessments as candidate surrogate endpoints in future interventional trials. While morphological parameters obtained by OCT showed only subtle changes, functional parameters obtained by OCT–A, ERG measurements and adaptometry may allow for the early detection of disease-modifying effects in response to systemic therapies aimed at restoring PPi homeostasis or preventing ectopic mineralization, even if it not known at the moment how the latter contributes to the findings reported here. One promising therapeutic strategy under investigation is *ENPP1* enzyme replacement therapy, which has shown potential in preclinical models and is currently being evaluated in clinical trials for GACI [19]. The observed stability of visual function over nearly a decade further supports the feasibility of such outcome measures in clinical studies. Moreover, identifying retinal biomarkers in *ENPP1* deficiency may extend to other ectopic calcification disorders, potentially enabling broader application of retinal endpoints in rare disease trials.

However, this study has several limitations that should be acknowledged. First, this is a single-case exploratory study, and the findings may not be generalizable across the broader phenotypic spectrum of *ENPP1* deficiency. Second, although retinal imaging and electrophysiological assessments were comprehensive, dark adaptation was assessed at only one time point because the required specialized equipment became available at our institution only late in the follow-up period. We will perform dark adaptation examinations in the future, as impaired dark adaptation has recently emerged as a sensitive biomarker in PXE-associated retinal pathology [5,20]. Third, potential confounding factors—including systemic comorbidities and long-term bisphosphonate therapy—may have influenced retinal structure or function. The influence of the complex medical history of the patient besides *ENPP1* deficiency on retinal blood flow and retinal function has to be evaluated in more detail in the future. This is particularly valid for retinal blood flow quantified by OCT–A, which may be hampered by the patient’s complex cardiovascular and hemodynamic history. Lastly, while reduced ERG amplitudes were observed, their clinical significance remains unclear in the absence of a clear progressive visual deterioration.

## 4. Materials and Methods

### 4.1. Acquisition and Collection of Human Subject Data

Human subject data were obtained within the framework of a retrospective, longitudinal natural history study of subjects with *ENPP1* deficiency or the early-onset form of ABCC6 deficiency (NCT05050669, https://clinicaltrials.gov, accessed on 30 April 2026). The study was approved by the ethics committee of the Medical Association of Westphalia-Lippe and Westphalian Wilhelm University (approval no. 2021-656-f-S). The proband provided written informed consent to participate in the study and gave permission to publish the results. All procedures were conducted in accordance with the Declaration of Helsinki.

All examinations were performed by qualified expert staff in our Eye Clinic, applying standardized examination protocols that are applied for all patients in our clinic and also for the healthy controls within this study. The same devices and protocols were used throughout the duration of this study. The timing and type of examination performed at each visit are summarized in Supplemental Table S4. The controls were healthy male and female volunteers recruited from staff and students at our clinic, broadly age-matched to the patient, with no known systemic or ophthalmologic diseases (see Supplemental Table S5). Control data for the single examination techniques were obtained from different healthy volunteers over several years in our clinic.

Both eyes of the patient and control participants were examined. To avoid treating fellow eyes as independent observations, measurements from the two eyes were averaged, yielding one representative value per control participant and one value per time point for the patient. Although this approach may obscure interocular asymmetry, differences between the two eyes were generally small and showed no consistent pattern over the observation period. For transparency, eye-specific VEP data are presented in Supplementary Figure S4.

### 4.2. Ophthalmological Inspection and Imaging

Best corrected visual acuity, intraocular pressure and overall ocular health were assessed.

**Figure 12:**
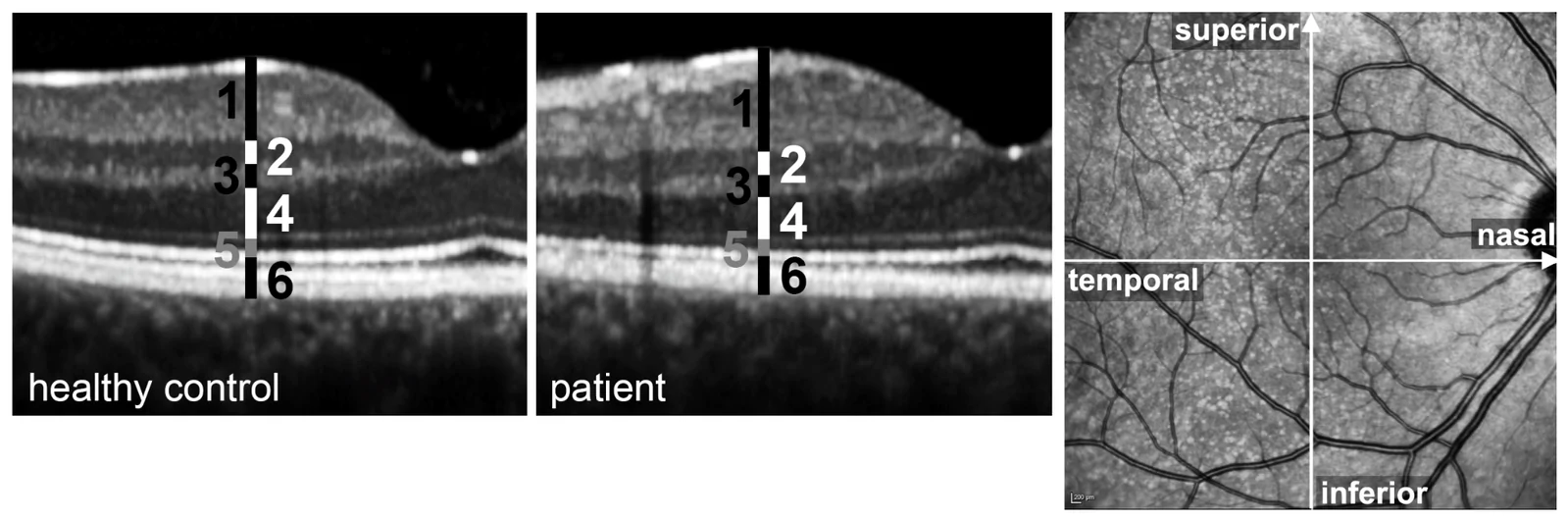
Demonstration of directions and choice of segmentation for the determination of retinal thickness. The numbers correspond to the following retinal layers: 1—nerve fiber layer, ganglion cell layer and inner plexiform layer (NFL–IPL), 2—inner nuclear layer (INL), 3—outer plexiform layer (OPL), 4—outer nuclear layer to the external limiting membrane (ONL–ELM), 5—from the external limiting membrane to the ellipsoid zone (ELM–EZ), 6—from the ellipsoid zone to the outer rim of the retinal pigment epithelium (EZ–RPE).

Fundus images were taken using a Zeiss Visucam 200 camera (Carl Zeiss Meditec, Oberkochen, Germany). Spectral-domain optical coherence tomography (SD-OCT), autofluorescence and infrared scanning laser ophthalmoscopy (IR-SLO) imaging as well as fluorescein angiography were performed using the "Spectralis" device (Heidelberg Engineering, Heidelberg, Germany). In OCT imaging, both macula and optic nerve head were inspected. Image quality was reproducible throughout the study. Due to the “eye tracking” mode of the device, there were no motion artifacts. Total retinal thickness was determined in OCT volume scans using the Heidelberg Engineering software HEYEX 2.0, which produced a thickness map, divided into sectors according to the ETDRS grid; the resulting data are used in Figure 4. The thicknesses of individual retinal layers were measured manually in the horizontal and vertical scan through the fovea centralis using the Heidelberg HEYEX 2.0 software. We measured thickness at a distance of 1 mm from the center of the fovea in all four directions using the “measure distance” tool of the Explorer software perpendicularly to the orientation of the retinal layers. The applied segmentation of the retina is demonstrated in Figure 12, and the results are shown in Figure 5.

Imaging of the retinal vasculature was performed by OCT angiography (OCT–A) using the RTVue XR Avanti with AngioVue (Optovue Inc., Fremont, CA, USA) and the Revue software (version 2017.1.0.151). The scan size was 3 × 3 mm^2^. The built-in AngioVue algorithm automatically performed segmentation and calculated flow density, which is given as a percentage value (%) for the different retinal layers and sublocations. Automated segmentation was performed using the device software AngioAnalytics version 2018.1.0.43. All scans were manually reviewed for segmentation errors and projection artifacts. The AngioVue tracking function automatically detects motion artifacts and initiates repeat acquisition when necessary. Scans with residual motion artifacts or missing data were excluded. Only images with a quality score of ≥7 and a signal strength index (SSI) of ≥50 were included. The device’s Auto Adjust mode was used to optimize image quality. However, axial length was not accounted for in the OCT–A analysis; therefore, potential effects of ocular magnification cannot be excluded and represent a limitation. The software also measured the size of the foveal avascular zone (FAZ) and the flow density in a radius of 300 µm around the center of the fovea (FAZ 300 µm flow density).

### 4.3. Electrophysiology

Electrophysiological measurements were performed according to the standard procedures recommended by the International Society for Clinical Electrophysiology of Vision (ISCEV) [22] using the Retiport/scan device by Roland Consult (Brandenburg, Germany).

Pattern visual evoked potentials (VEP) were recorded with best visual acuity by presenting a checkerboard pattern on a TFT monitor at a resolution of first 1° and then 0.25°, i.e., 15′. VEP were recorded using a gold electrode attached to the inion, with counter and ground electrodes attached to the nasion and oz, respectively, and elicited by switching the checkerboard squares between black and white at a frequency of 1.4 Hz.

For electroretinography (ERG), the pupils of the patient’s eyes were dilated by triplicate application of Mydriaticum Stulln drops over 30 min, and pupil dilation was checked by the performing technician. Corneal desensitization was achieved by a drop of proparacaine. Retinal potentials were recorded by DTL electrodes. First, photopic (light-adapted) measurements were performed, i.e., photopic flash ERG and 30 Hz Flicker ERG, both at a light intensity of 3.0 cd·s/m^2^. The patient was then dark-adapted over 30 min, and scotopic flash ERG measurements were performed at light intensities of 0.01 cd·s/m^2^, 3.0 cd·s/m^2^ and 100 cd·s/m^2^; the latter was used to record isolated oscillatory potentials at filter settings changed to high frequencies. Multifocal ERG was recorded in the same set-up under light-adapted conditions by stimulation with a 61-field hexagon pattern with a TFT monitor.

### 4.4. Dark Adaptometry

The commercial DARK-adaptometer Mark 4 device (Roland Consult, Brandenburg, Germany) was used to perform dark adaptometry. Stimulation was performed with green or red light at a temporal eccentricity of 30°. Due to the limited endurance of the patient, the standard protocol was modified. Photoreceptor bleaching period by bright illumination was shortened to 45 s, and total dark adaptation time was 25 min. We recorded sensitivity of the right eye. Both the patient and the age-matched healthy controls underwent the same protocol to ensure comparability of the results.

### 4.5. Statistics

As we performed a single-case study with only one patient, statistical examination was performed according to the approach described by Crawford and Howell (1998) that uses a modified *t*-test [21]. Calculations were performed using the software singslims_ES.exe (https://homepages.abdn.ac.uk/j.crawford/pages/dept/SingleCaseMethodsComputerPrograms.HTM, access date on 20 April 2026), provided by J.R. Crawford et al. [23], using the mean, standard deviation and sample number of a control group and the value of the single patient. As the latter, we used the mean of the values obtained during the observation method.

Because only one affected individual was examined, statistical comparisons with controls should be interpreted as single-case comparisons against normative reference data and should not be generalized to the ENPP1-deficient population. Longitudinal changes within the patient are primarily interpreted descriptively.

We used GraphPad Prism 11 for the graphical presentation of the data. Tukey-style box-and-whisker plots (named Tukey box plots in the figure captions) presented control data and single-dot plots data of the patient. In the Tukey box plots, the boxes represent the interquartile range of the data, the line inside the box is the median value, and the whiskers have the length of 1.5 × the interquartile range. All data points outside the boundary of the whiskers are plotted as outliers. Preparation of the diagrams was completed using CorelDraw 2020, version 22.1.1.523.

## 5. Conclusions

This longitudinal case study identified subtle structural, microvascular and functional retinal abnormalities in one individual with molecularly confirmed *ENPP1* deficiency despite relatively preserved visual function. These observations generate hypotheses regarding possible subclinical retinal involvement and identify multimodal ophthalmological measures that warrant validation in larger longitudinal ENPP1-deficient cohorts.

## Data Availability

The raw data supporting the conclusions of this article will be made available by the authors on request.

## Supplementary Materials

The following supporting information can be downloaded at: https://www.mdpi.com/article/10.3390/ijms27188401/s1.

## Abbreviations

The following abbreviations are used in this manuscript:

ABCC6	ATP-binding cassette subfamily C member 6
ChC	choriocapillaris
ELM	external limiting membrane
ENPP1	ectonucleotide pyrophosphatase/phosphodiesterase 1
ERG	Electroretinography; electroretinographic
ETDRS	Early Treatment Diabetic Retinopathy Study
EZ	ellipsoid zone
FAZ	foveal avascular zone
GACI	generalized arterial calcification of infancy
INL	inner nuclear layer
IPL	inner plexiform layer
IR-SLO	Infrared scanning laser ophthalmoscopy
NFL	nerve fiber layer
OCT	optical coherence tomography
OCT–A	optical coherence tomography angiography
OD	oculus dexter (right eye)
ONL	outer nuclear layer
OPL	outer plexiform layer
OS	oculus sinister (left eye)
PP_i_	pyrophosphate
PXE	Pseudoxanthoma elasticum
RPE	retinal pigment epithelium
VEP	visually evoked potential
