# Supplementary material for: Retinal Features and Preserved Vision in ENPP1 Deficiency: Long-Term Insights from Multimodal Imaging and Electrophysiology
Source: Int J Mol Sci. 2026 Sep 21;27(18):8401. doi: 10.3390/ijms27188401 (PMC13607652; doi:10.3390/ijms27188401)
Supplement: Supplementary file 1 [file ijms-27-08401-s001.zip › Supplemental tables.pdf]

Tables S1-S3: Electrophysiological measurements from the patient were first averaged across both eyes at each visit and are reported as mean ± SD across all available visits between 2013 and 2026. Corresponding reference values obtained from healthy volunteers examined at our clinic are presented as mean ± SD for comparison.

Table S1: Flash ERG:

mode	latency [ms]		amplitude [µV]	
	patient	control range	Patient	control range
scotopic 0.01 b-wave	80.2 ± 6.4	73.4 ± 4.5	197 ± 82	302 ± 43
scotopic 3.0 a-wave	17.2 ± 1.9	16.0 ± 0.4	169 ± 48	307 ± 48
scotopic 3.0 b-wave	43.5 ± 5.2	46.0 ±3.7	318 ± 99	548 ± 58
scotopic oscill. potentials	22.7 ± 0.5	21.6 ± 0.5	65.8 ± 18.5	84.8 ± 24.0
scotopic b/a ratio			1.86 ± 0.19	1.81 ± 0.21
photopic 3.0 a-wave	15.1 ± 0.3	14.5 ± 0.6	30.6 ± 13.8	55.0 ±13.1
photopic 3.0 b-wave	32.1 ± 2.4	30.4 ±1.5	155 ± 54	243 ± 61
photopic 30 Hz Flicker	34.0 ± 0.6	31.7 ± 1.5	64.4 ± 18.9	76.8 ± 13.1

Table S2: Multifocal ERG:

no. of ring	latency [ms]		amplitude [nV/deg²]	
	patient	control range	patient	control range
1. ring	40.5 ± 3.0	38.7 ± 2.0	113 ± 29	187 ± 34
2. ring	35.3 ± 1.8	36.0 ± 2.2	61.6 ± 13.8	91.9 ±14.4
3. ring	37.8 ± 2.8	34.8 ± 2.2	34.3 ± 8.2	56.2 ± 8.1
4. ring	38.7 ± 2.3	34.9 ± 2.5	26.8 ± 7.2	39.2 ± 5.2
5. ring	38.3 ± 1.9	35.3 ± 2.7	20.5 ± 6.2	29.8 ± 4.5

Table S3: Pattern VEP:

resolution	latency [ms]		amplitude [µV]	
	**patient**	**control range**	**patient**	**control range**
1°	101 ± 3	104 ± 5	14.4 ± 2.0	18.1 ± 5.6
0.25°	107 ± 2	107 ± 5	16.1 ± 2.1	20.3 ± 6.8

Table S4: List of timepoints of *in-vivo* imaging and functional testing of the patient

date	Age of patient [years]	VA	Color fun-dus	OCT retina	OCT BMO	OCT-A	ERG	VEP	DA
09. 12. 2013	16.93	✓					✓		
07. 08. 2014	17.59	✓							
26. 02. 2016	19.15	✓	✓	✓					
11. 09. 2017	20.69	✓	✓	✓					
04. 12. 2018	21.92	✓	✓	✓	✓				
22. 03. 2021	24.22	✓	✓	✓	✓				
30. 11. 2021	24.91		✓	✓	✓		✓	✓	
13. 01. 2023	26.03	✓	✓	✓	✓		✓	✓	
13. 09. 2023	26.70			✓	✓	✓			
13. 03. 2024	27.20	✓	✓	✓	✓	✓	✓	✓	
27. 09. 2024	27.74	✓	✓		✓		✓	✓	
28. 03. 2025	28.24		✓	✓	✓	✓	✓	✓	
09. 10. 2025	28.78	✓		✓	✓	✓	✓	✓	
23. 02. 2026	29.15		✓	✓	✓	✓	✓	✓	✓

Abbreviations: VA - visual acuity, OCT – optical coherence tomography, BMO - Bruch's membrane opening, OCT-A – OCT angiography, ERG – electroretinography, VEP – visual evoked potentials, DA – dark adaptometry

Table S5: Description of control groups

Figure / parameter	number	age: median (percentiles)	male/ female	used eyes (right/left)
Figs. 4 and 5: retinal thickness ETDRS and thickness of retinal layers	14	26 (23; 27.5)	7 / 7	12 / 11
Fig. 6: thickness of retinal NFL	11	27 (23; 29)	5 / 6	11 / 10
Fig. 7: OCT-A parameters	29	25 (24; 28.5)	18 / 11	27 / 17
Fig. 9: ERG parameters (flash and multifocal, 17 individuals the same)	17<br>18	23 (19.5; 27)<br>21 (19.8; 29.8)	2 / 15<br>2 / 16	16 / 16<br>16 / 15
Fig. 10: VEP parameters	41	23 (23; 25)	7 / 34	40 / 36
Fig. 11: Dark adaptometry	6	25.5 (22.8; 29.2)	2 / 4	6 / 0
